# Trends in the growing impact of group A *Streptococcus* infection on public health after COVID-19 pandemic: a multicentral observational study in Okayama, Japan

**DOI:** 10.1007/s10096-024-05015-2

**Published:** 2024-12-16

**Authors:** Shinnosuke Fukushima, Takashi Saito, Yoshitaka Iwamoto, Yuko Takehara, Haruto Yamada, Koji Fujita, Masayo Yoshida, Yasuhiro Nakano, Hideharu Hagiya

**Affiliations:** 1 Department of General Medicine, Okayama University Graduate School of Medicine, Dentistry and Pharmaceutical Sciences, Okayama, Japan; 2 Department of Bacteriology, Okayama University Graduate School of Medicine, Dentistry and Pharmaceutical Sciences, Okayama, Japan; 3 Department of Infectious Diseases, Okayama University Hospital, 2-5-1 Shikata-cho, Okayama, Okayama 700-8558 Japan; 4 Department of General Medicine, NHO Okayama Medical Center, Okayama, Japan; 5 Department of Surgery, Okayama Saiseikai General Hospital, Okayama, Japan; 6 Department of General Medicine, Okayama City Hospital, Okayama, Japan; 7 Department of General Medicine and Infectious Diseases, Tsuyama Chuo Hospital, Okayama, Japan; 8 Department of General Medicine, Okayama Kyoritsu Hospital, Okayama, Japan

**Keywords:** Epidemiology, Group A *Streptococcus*, Necrotizing fasciitis, Streptococcal toxic shock syndrome, Surveillance

## Abstract

**Purpose:**

Following the COVID-19 pandemic, group A *Streptococcus* (GAS) infection has been surging worldwide. We aimed to compare the disease burden between notified cases of streptococcal toxic shock syndrome (STSS) and unreported GAS infections.

**Methods:**

This is a multicentral observational study, retrospectively performed at seven hospitals in Okayama prefecture in Japan from January 2022, to June 2024. Clinical and microbiological data of patients with positive cultures of GAS were collected from the medical records. Primary outcomes were defined as rates of surgical procedures, intensive care unit (ICU) admission, and in-hospital mortality, which were compared among patients with locally-defined STSS, invasive GAS (iGAS), and non-iGAS infection.

**Results:**

GAS was detected in 181 patients, with 154 active cases of GAS infection. The number of patients with GAS infection surged in late 2023. The most common source of infection was skin and soft tissue infections, accounting for 83 cases, including 15 cases of necrotizing fasciitis, and 12 cases (7.8%) were notified to public health authorities as STSS. Among the 25 unreported iGAS cases, 9 (36.0%) underwent surgical intervention, and 4 patients (16.0%) required ICU admission. The mortality rates in the unreported iGAS cases were comparable to those observed in the notified STSS.

**Conclusions:**

We highlighted that the number of iGAS infections was twofold higher than that of notified STSS, with comparable mortality rate between these groups, indicating substantial underestimation of the true burden of iGAS. This epidemiological investigation has significant implications for enhancing infectious disease surveillance frameworks and public health policy development.

## Introduction

Streptococcal toxic shock syndrome (STSS) is a life-threatening infectious disease characterized by the rapid onset of systemic symptoms and progression to multiple organ failures [1, 2]. Different from the conventional definition of STSS, Japanese surveillance protocols for STSS encompass infections caused not only by group A *Streptococcus* (GAS, *Streptococcus pyogenes*), but also by group B, C, and G β-hemolytic streptococci [3]. Notably, mortality rates associated with GAS infections remain substantially high, despite appropriate antimicrobial therapy and surgical interventions. In response to this significant public health concern, comprehensive international surveillance systems for invasive GAS (iGAS) infections have been established globally [4–11].

GAS is classified based on the *emm* gene sequence, which encodes the M protein, a well-known virulence factor [12]. Recently, the *emm*1 type (M1) strain was reported to be the most frequently isolated strain from upper respiratory tract specimens in patients with pharyngitis, scarlet fever, and invasive infections in the United Kingdom [13]. Since 2011, a highly pathogenic *emm1* type GAS strain, which produces approximately nine times more erythrogenic toxin and exhibits higher transmissibility [14], later termed the “M1UK lineage”, has emerged and become predominant among M1 strains isolated in Europe and Australia [9, 10, 13–15]. ​​This M1UK strain has recently been identified as a cause of STSS in Japan, with reports of its association with respiratory tract infections [16, 17].

In Japan, the number of patients with locally-defined STSS is monitored as a category V notifiable infectious disease under the Infectious Disease Epidemiology Surveillance [18]. Notification is legally required if specific criteria are met, including a state of systemic shock, liver failure, renal failure, acute respiratory distress syndrome, disseminated intravascular coagulation, soft tissue inflammation, generalized erythematous rash, and central nervous system symptoms, and the detection of β-hemolytic streptococci in sterile samples such as blood [19]. Although the result of the STSS surveillance is open to the public [16, 20] updates are infrequent, and detailed clinical information is lacking. Additionally, patients with severe conditions necessitating intensive care unit (ICU) admission or surgical intervention are not captured by the current surveillance system if they fail to fulfill the established diagnostic criteria for STSS. Consequently, the clinical burden of GAS infections may be largely underestimated within the current reporting framework. Therefore, our objective is to elucidate the true impact of GAS infections in Japan, particularly amidst the recent surge of GAS infections.

## Methods

### Study design and settings

We retrospectively collected the clinical and microbiological data from patients with positive cultures of GAS from the medical and microbiological records at seven hospitals across three municipalities in Okayama Prefecture, Japan (Okayama University Hospital, NHO Okayama Medical Center, Okayama Saiseikai General Hospital, Okayama City Hospital, Tsuyama Chuo Hospital, Okayama Kyoritsu Hospital, Takahashi Central Hospital), between January 1, 2022, and June 30, 2024. Five institutions (Okayama University Hospital, NHO Okayama Medical Center, Okayama Saiseikai General Hospital, Okayama City Hospital, Okayama Kyoritsu Hospital) were located in Okayama City (population approximately 700,000). Tsuyama Chuo Hospital and Takahashi Central Hospital cover Tsuyama City (population approximately 100,000) and Takahashi City (population approximately 29,000), respectively, based on 2020 census data. Among participating institutions, Okayama University Hospital and Tsuyama Chuo Hospital were designated tertiary care hospitals, while the others were secondary acute care hospitals. Ethical approval was obtained from the Institutional Review Board of Okayama University Hospital (No. 2406-015). The requirement for informed consent was waived due to the retrospective nature of the study, which involved the use of fully anonymized, routinely collected data.

### Inclusion criteria

We included cases of patients with positive cultures of GAS from any specimen during the study period. Episodes with intervals of three months or more were defined as different cases.

### Definitions and study protocol

The patients diagnosed with GAS were classified as either infectious or carrier cases. STSS is a notifiable disease classified as a category V infectious disease that must be reported upon diagnosis [18]. Thus, when patients with this condition, medical practitioners are obligated to inform local public health centers using reporting documents. Since April 2006, all cases of the locally-defined STSS caused by β-hemolytic streptococci are supposed to be reported as category V infectious diseases.

We retrospectively collected the following information from patient medical records: age, sex, time of diagnosis, underlying conditions, specimen type from which GAS was detected, clinical diagnosis, notification status as a category V infectious disease, surgical procedures performed, admission to the ICU, and in-hospital mortality. Age groups were categorized in 10-year increments. To analyze the temporal trend of GAS infections, we divided the study period into six-month intervals: January–June in 2022, July–December in 2022, January–June in 2023, July–December in 2023, and January–June in 2024.

A patient was defined as a GAS carrier if the bacteria were detected at a body site other than the focus of infection or if the attending physician determined that the patient was a carrier. We categorized the infectious foci into six groups: pharyngitis, lower respiratory infections, skin and soft tissue infections (SSTIs), abdominal and pelvic infections, genital infections, and other infections. The infectious foci were determined in most cases by the attending physician based on a match between the site of infection and the site of an obtained specimen. The pharyngitis category encompasses peritonsillar abscess cases confirmed through microbiological cultures from pharyngeal specimens or purulent material obtained from peritonsillar collections. The iGAS infections were defined according to the United States Centers for Disease Control and Prevention (CDC)’s Active Bacterial Core surveillance criteria, comprising cases of STSS, necrotizing fasciitis, and isolation of GAS from normally sterile anatomical sites, including but not limited to blood, cerebrospinal fluid, synovial fluid, peritoneal fluid, osseous tissue, and internal organs [4, 5].We investigated several comorbidities, including hypertension, cardiovascular disease, diabetes mellitus, malignancy, skin disorder, chronic obstructive pulmonary disease or asthma, kidney disorder, liver disorder, mental disorder, and others. Whole-genome sequence analysis of GAS strains isolated from notified cases commenced in January 2024 in Japan, prompted by the increased incidence of GAS infections and the potential emergence of the M1UK in 2023. We also gathered this data from the participating hospital.

### Outcome measures and statistical analysis

The primary outcomes assessed included the difference of ICU admission, the need for surgical intervention, notification as a Category V infectious disease, and in-hospital mortality between patients with notified STSS cases and unreported cases. The secondary outcome focused on the difference between SSTI and other cases. Continuous variables were presented as medians with interquartile ranges (IQRs) and evaluated using the Kruskal–Wallis test or Mann–Whitney U test. Categorical variables were reported as numbers and percentages and were assessed using the Chi-square or the Fisher’s exact test. Data analysis was conducted using EZR software, a graphic user interface for the R 3.5.2 software (The R Foundation for Statistical Computing, Vienna, Austria). All reported *p* values < 0.05 were considered statistically significant.

## Results

### Backgrounds and details of the eligible cases

**Fig. 1:**
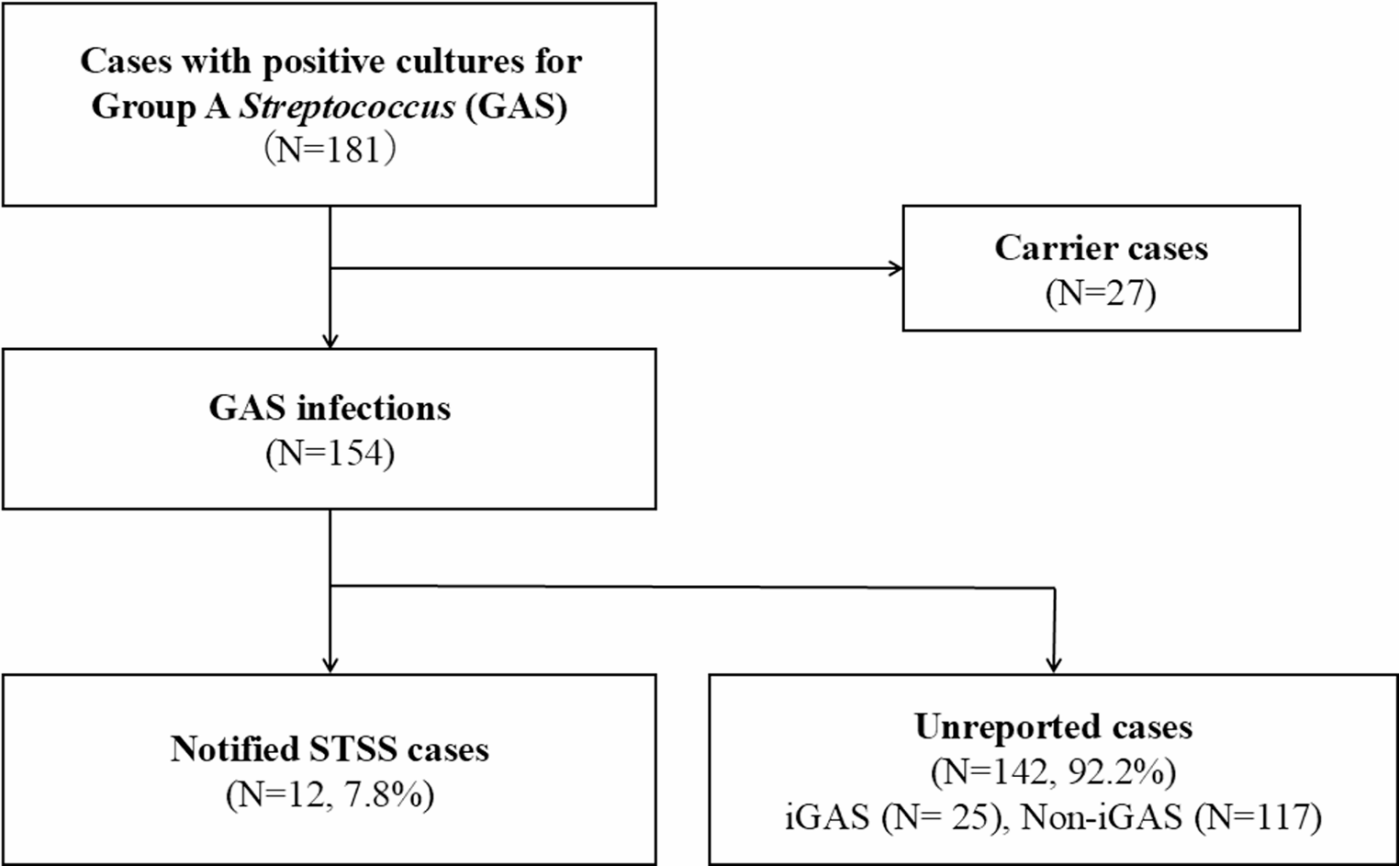
Study enrolment flow. GAS: group A *streptococcus*, iGAS: invasive GAS, STSS: streptococcal toxic shock syndrome. Of the 154 GAS infection cases, 12 cases (7.8%) were notified as STSS cases, whereas the remaining 142 cases (92.2%) did not meet the diagnostic criteria for STSS. Among the unreported cases, invasive cases are 25, 117 are non-invasive cases

**Fig. 2:**
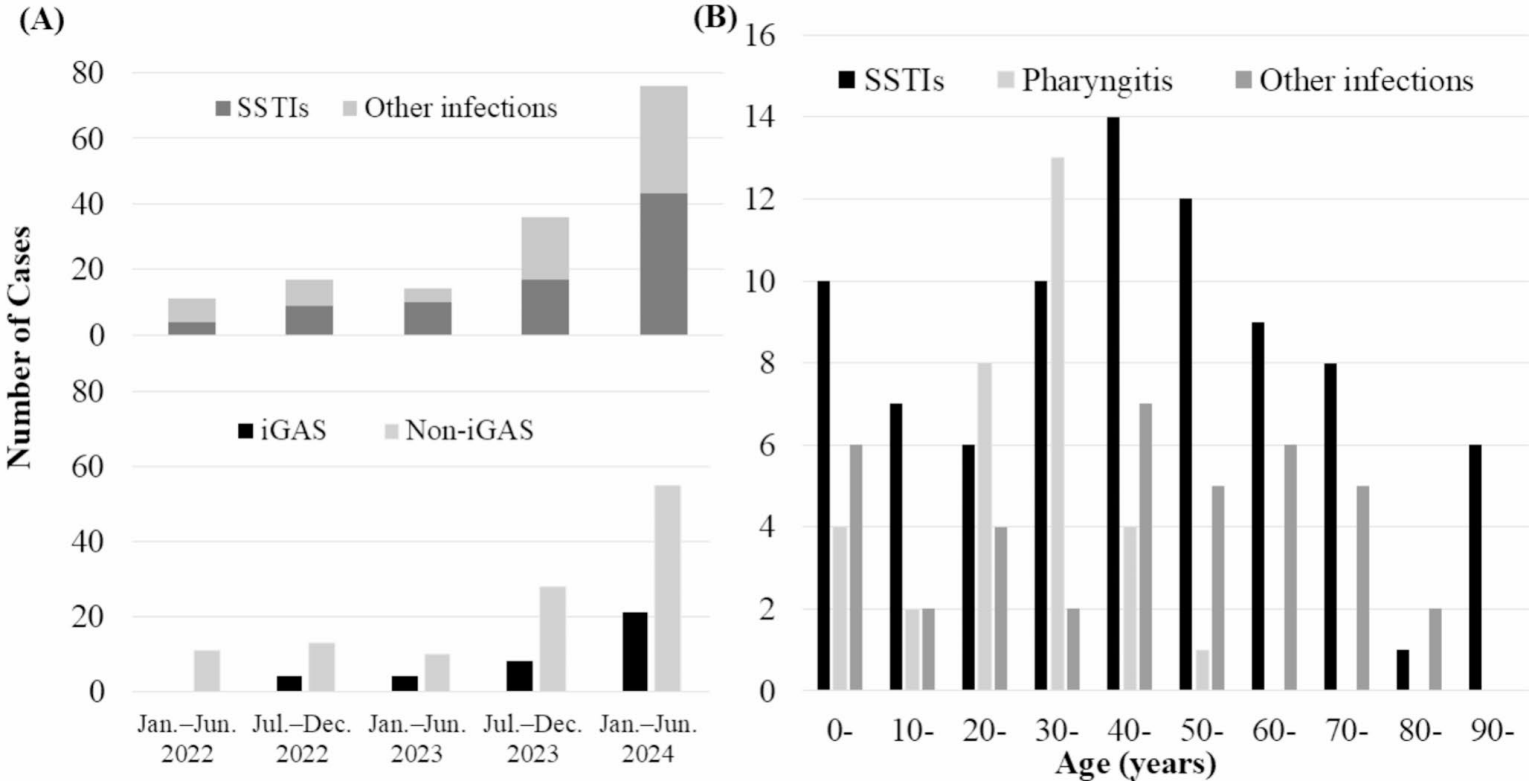
Number of patients diagnosed with GAS infection, (**A**) by six-month intervals and (**B**) by patient age. GAS: group A *streptococcus*, iGAS: invasive GAS, SSTI: skin and soft tissue infection. Other infections include pharyngitis, respiratory and ear infections, abdominal and pelvic infections, genital infections, parotitis, conjunctivitis, primary bacteremia, and streptococcal toxic shock syndrome

A total of 181 patients had positive cultures for GAS during the study period, with a median age of 43 years [IQR, 19–67] (Table 1). Of the total isolates, 27 represented asymptomatic colonization, while 154 cases were confirmed as active GAS infections. The clinical manifestations comprised 32 cases (17.7%) of pharyngitis, 7 cases (3.9%) of lower respiratory infections, and 83 cases (45.9%) of SSTIs, including 15 cases of necrotizing fasciitis. Among these infectious cases, 37 cases met the diagnostic criteria for iGAS infections. GAS was isolated from 194 specimens collected from these 181 patients, with the most frequent source being pus (41.2%), followed by pharynx (11.3%) and blood (9.8%). Specimens obtained from normally sterile anatomical sites constituted 23 cases (11.9%) of the total isolates, comprising blood cultures, synovial fluid aspirates, and peritoneal fluid specimens. Of the 154 GAS infection cases, 12 cases (7.8%) were notified to public health authorities as STSS cases. The remaining 142 cases (92.2%) were unreported, including 25 iGAS cases (Fig. 1). The number of patients with GAS infection ranged 10–20 cases per half-year from 2022 to the first half of 2023, and increased to 36 cases from July to December 2023 and surged to 76 cases from January to June 2024 (Fig. 2A). Patient ages varied from children to middle-aged population (Fig. 2B).

**Table 1:** Clinical backgrounds of patients isolated with GAS and breakdown of GAS-positive specimens

**Number of patients (total)**	181
**Age**,** years (median [IQR])**	43 [19–67]
**Sex**,** male (%)**	110 (60.8)
**Clinical diagnosis (%)**	
Carrier state	27 (14.8)
Pharyngitis	32 (17.7)
Lower respiratory infections	7 (3.9)
Skin and soft tissue infections	83 (45.6)
Necrotizing fasciitis	15 (8.2)
Abdominal and pelvic infections	8 (4.4)
Genital infections	6 (3.3)
Other infections*	18 (9.9)
iGAS infections (%)	37 (20.4)
**Specimen (%)**	
Total	194
Sterile site specimens	23 (11.9)
Blood	19 (9.8)
Synovial fluid	2 (1.0)
Ascites	2 (1.0)
Non-sterile site specimens	171 (88.1)
Pus	80 (41.2)
Pharynx	22 (11.3)
Sputum	15 (7.7)
Skin	15 (7.7)
Eye discharge	9 (4.6)
Vaginal discharge	8 (4.1)
Urine	6 (3.1)
Exudate	5 (2.6)
Others**	11 (5.7)

GAS: group A *streptococcus*, iGAS: invasive GAS, IQR: interquartile range

*Other infections include primary bacteremia (5), ear infections (4), streptococcal toxic shock syndrome (2), conjunctivitis (2), lymphadenitis (2), parotitis (1), poststreptococcal glomerulonephritis (1), and unknown (1)

**Others include ear and nasal discharge (4), tissue (2), wound swabs (2), vulva and uterus (2), and feces (1)

### Clinical features and outcomes in GAS infections

#### Summarization of all cases

Overall details for the GAS infections are presented in Table 2. The median age of 154 patients with GAS infections was 43 years (IQR, 19–67 years), and 60.4% were male. Nearly half (54.5%) of them had no underlying comorbidities. Clinical diagnosis of GAS infection varied, with SSTIs (53.9%), pharyngitis (20.8%), other infections (7.8%), abdominal and pelvic infections (5.2%), lower respiratory infections (4.5%), and genital infections (3.9%). Of all, 19 (12.3%) developed GAS bacteremia, 36 (23.4%) required surgical interventions, 19 (12.3%) were admitted to ICU, and 3 (1.9%) died in the hospitals. The prognosis of two patients was unknown due to transfer to other hospitals. Six cases met the diagnostic criteria for the locally-defined STSS and were notified to the public health authorities in 2024. The M1UK lineage strains were identified in 66.7% (4 out of 6 cases) of these cases, while the remaining cases were attributed to *emm*81 and *emm*89 genotypes, with one case each.

**Table 2:** Comparative analysis of clinical backgrounds, manifestations, and outcomes across the three spectrum of GAS infections: notified STSS cases, unreported iGAS cases, and unreported non-iGAS cases

	Overall		Notified cases		Unreported cases				*p* value	
			STSS		All GAS cases	iGAS	Non-iGAS		STSS vs. iGAS	STSS vs. Non-iGAS
**Number of cases (total)**	154		12		142	25	117			
**Age**,** years (median [IQR])**	43 [19–67]		60 [42–78]		39 [15–63]	51 [29–73]	37 [15–59]		0.63	< 0.001***
**Sex**,** male (%)**	93 (60.4)		6 (50.0)		87 (61.3)	14 (56.0)	73 (62.4)		1	0.54
**Underlying clinical conditions (%)**										
Presence of comorbidity	70 (45.5)		9 (75.0)		61 (43.0)	18 (72.0)	43 (36.8)		1	0.014***
**Clinical diagnosis (%)**										
Pharyngitis	32 (20.8)		0 (0.0)		32 (22.5)	0 (0.0)	32 (27.4)		-	0.037***
Lower respiratory infections	7 (4.5)		0 (0.0)		7 (4.9)	0 (0.0)	7 (6.0)		-	1
Skin and soft tissue infections	83 (53.9)		8 (66.7)		75 (52.8)	18 (72.0)	57 (48.7)		1	0.36
Necrotizing fasciitis	15 (9.7)		4 (33.3)		11 (7.7)	11 (44.0)	0 (0.0)		0.72	< 0.001***
Abdominal and pelvic infections	8 (5.2)		2 (16.7)		6 (4.2)	0 (0.0)	6 (5.1)		0.099	0.16
Genital infections	6 (3.9)		0 (0.0)		6 (4.2)	1 (4.0)	5 (4.3)		1	1
Other infections*	18 (11.7)		2** (16.7)		16 (11.3)	5 (20.0)	11 (9.4)		1	0.35
iGAS infections	37 (24.0)		12 (100)		25 (17.6)	25 (100)	0 (0.0)		-	< 0.001***
**Complication (%)**										
Bacteremia	19 (12.3)		7 (58.3)		12 (8.5)	12 (48.0)	0 (0.0)		0.73	< 0.001***
**Diagnosed year (%)**										
2022	28 (18.2)		1 (8.3)		27 (19.0)	3 (12.0)	24 (20.5)		1	0.46
2023	50 (32.5)		5 (41.7)		45 (31.7)	7 (28.0)	38 (32.5)		0.47	0.53
2024 (January–June)	76 (49.4)		6 (50.0)		70 (49.3)	15 (60.0)	55 (47.0)		0.73	1
**Outcomes (%)**										
Surgical interventions	36 (23.4)		11 (91.7)		25 (17.6)	9 (36.0)	16 (13.7)		0.0018***	< 0.001***
Intensive care unit	19 (12.3)		11 (91.7)		8 (5.6)	4 (16.0)	4 (3.4)		< 0.001***	< 0.001***
In-hospital mortality	3 (1.9)		1 (8.3)		2 (1.4)	2 (8.0)	0 (0.0)		1	0.093

GAS: group A *streptococcus*, iGAS: invasive GAS, STSS: streptococcal toxic shock syndrome, IQR: interquartile range

*Other infections include ear infections, lymphadenitis, parotitis, conjunctivitis, primary bacteremia, and streptococcal toxic shock syndrome

**Two cases were diagnosed with bacteremia and streptococcal toxic shock syndrome

***Significant differences are observed

#### Notified STSS cases vs. unreported iGAS cases

Among the unreported cases, iGAS infections accounted for 25 cases (17.6%). No statistically significant differences were observed in age and gender distribution, comorbidity prevalence, clinical diagnoses, annual case distribution, or complications. Although patients with STSS underwent surgical interventions more frequently (91.7% vs. 36.0%; *p* = 0.0018) and demonstrated higher rates of ICU admission (91.7% versus 16.0%; *p* < 0.001), in-hospital mortality rates were comparable between STSS cases and unreported iGAS cases (8.3% vs. 8.0%).

#### Notified STSS cases vs. unreported non-iGAS cases

Among the unreported cases, non-iGAS infections constituted 117 cases (76.0%) of the total cohort. Patient with the notified STSS cases were significantly older (60 years vs. 37 years; *p* < 0.001). No significant difference was observed in the proportions of male and female patients. The proportion of patients with underlying diseases was significantly higher among those with the notified STSS cases (75.0% vs. 36.8%; *p* = 0.038). There were no cases of pharyngitis, lower respiratory infections among the notified STSS cases. Both the numbers of notified STSS cases and unreported non-iGAS cases increased year by year, with no significant difference noted between the two groups. Patients with the notified STSS cases underwent surgical interventions more frequently (91.7% vs. 13.7%; *p* < 0.001) and had a higher rate of ICU admission (91.7% vs. 3.4%; *p* < 0.001). Although the difference was not statistically significant, in-hospital mortality was higher among patients with the notified STSS cases (8.3% vs. 0%; *p* = 0.093).

#### SSTIs vs. pharyngitis vs. other infections

The clinical characteristics of patients with SSTIs, pharyngitis, and other infections are given in Table 3. SSTIs accounted for 83 cases (53.9%), 32 (20.8%) are pharyngitis, and 39 (25.3%) are other infections. Significant differences were observed in the ages of the three groups (46 years vs. 33 years vs., 47 years) or in the proportions of male patients (65.1% vs. 68.8% vs. 43.6%). However, the prevalence of underlying diseases was significantly higher in SSTI cases for hypertension (*p* = 0.017), cardiovascular disease (*p* = 0.017), and skin disorders (*p* = 0.035). The skin disorders included 12 cases of atopic dermatitis and 2 cases of psoriasis vulgaris. When compared among three GAS infections, there were no significant differences in the proportions of STSS, the need for surgical interventions, and in-hospital mortality. The ICU admission rates of pharyngitis was significantly lower than other groups.

**Table 3:** The clinical characteristics of skin and soft tissue infections (SSTIs), pharyngitis, and other infections

	Overall	SSTIs	Pharyngitis	Other infections	*p* value
**Number of cases (total)**	154	83 (53.9)	32 (20.8)	39 (25.3)	
**Age**,** years (median [IQR])**	43 [19–67]	46 [21–71]	33 [20–46]	47 [22–72]	0.0017**
**Sex**,** male (%)**	93 (60.4)	54 (65.1)	22 (68.8)	17 (43.6)	0.049**
**Underlying clinical conditions (%)**					
No comorbidity	84 (54.5)	39 (47.0)	28 (87.5)	17 (43.6)	< 0.001**
Hypertension	15 (9.7)	13 (15.7)	0 (0.0)	2 (5.1)	0.017**
Cardiovascular diseases	12 (7.8)	11 (13.3)	1 (3.1)	0 (0.0)	0.017**
Diabetes mellitus	13 (8.4)	8 (9.6)	1 (3.1)	4 (10.3)	0.50
Malignancy	9 (5.8)	5 (6.0)	0 (0.0)	4 (10.3)	0.19
Skin disorders	14 (9.1)	12 (14.5)	0 (0.0)	2 (5.1)	0.035**
COPD or asthma	9 (5.8)	5 (6.0)	0 (0.0)	4 (10.3)	0.19
Kidney disorders	3 (1.9)	2 (2.4)	0 (0.0)	1 (2.6)	1
Liver disorders	5 (3.2)	1 (1.2)	1 (3.1)	3 (7.7)	0.12
Mental disorders	6 (3.9)	3 (3.6)	1 (3.1)	2 (5.1)	0.86
Others*	13 (8.4)	8 (9.6)	1 (3.1)	4 (10.3)	0.50
**Outcomes (%)**					
Class V notification (STSS)	12 (7.8)	8 (9.6)	0 (0.0)	4 (10.3)	0.18
Surgical interventions	36 (23.4)	20 (24.1)	11 (34.4)	5 (12.8)	0.11
Intensive care unit	19 (12.3)	13 (15.7)	0 (0.0)	6 (15.4)	0.033**
In-hospital mortality	3 (1.9)	2 (2.4)	0 (0.0)	1 (2.6)	1

GAS: group A *streptococcus*, IQR: interquartile range, COPD: chronic obstructive pulmonary disease, STSS: streptococcal toxic shock syndrome

*Others include Down syndrome, dyslipidemia, hyperuricemia, Hashimoto’s disease, rheumatoid arthritis, chronic sinusitis, uterine fibroids, and endometriosis

**Significant differences are observed among three groups

## Discussion

We herein described the clinical backgrounds and outcomes of patients diagnosed with GAS infections ranging from January 2022 to June 2024. During the two and half years, we obtained data for 154 GAS infections at seven Japanese hospitals. The number of patients with GAS infection increased starting in July 2023, with 12 notified cases of STSS. The most common sites of infection were SSTIs (83 cases), including 15 cases of necrotizing fasciitis. Clinical outcomes of GAS infections were overall severe, with regard to the requirement for surgical interventions (23.4%), ICU admission (12.3%), and in-hospital mortality (1.9%). The number of unreported iGAS cases was twofold higher than that of the notified STSS cases (25 cases vs. 12 cases), indicating substantial underestimation of the true clinical burden of invasive streptococcal disease within the current surveillance system. Nearly one-fifth of unreported GAS cases underwent surgical interventions, and 5.7% of the cases were managed in the ICU, with two ultimately resulting in in-hospital deaths. These findings underscore the clinical significance of unreported GAS cases that do not meet the STSS notification criteria.

STSS is the most severe form of streptococcal infection, with a mortality rate of 15.7–40.9%, even when appropriate antimicrobial therapy and surgical debridement are given to the patients [1, 21, 22]. It is commonly complicated by bacteremia and necrotizing fasciitis, necessitating early diagnosis and intensive support for organ failures in the ICU [2, 23]. GAS infections develop STSS more frequently than other streptococci, with an odds ratio of 3.28 (95% confidence interval: 1.21–8.77) [3]. Among patients suffering from iGAS infections, the likelihood of developing STSS ranges from 8 to 14% [24]. Many countries, including the United States and Europe, are investigating the spread and epidemiology of GAS infections through surveillance [4, 5, 7, 9–11]. Our study identified 37 iGAS infections, among which notified STSS cases constituted 32.4%, representing a higher proportion than previously documented in the literature [24]. In our study, 8.4% of patients with GAS infections were notified as Category V infectious diseases under the diagnosis of STSS, with an in-hospital mortality rate of 8.3% in notified STSS and 8.0% in unreported iGAS cases. Although these mortality rates are lower than previously reported [3, 21], our analysis was limited to inpatient mortality at each hospital and could not capture deaths occurring after inter-hospital transfer. Furthermore, the incidence of STSS is underestimated due to the stringent notifying criteria for STSS in Japan; indeed, the frequency of unreported iGAS infections was twofold higher than that of reported STSS cases. Similar to practices in other countries, it may be beneficial to include all the iGAS cases in reporting to prove necessary public health alerts.

The M1UK lineage strains among GAS have attracted worldwide attention due to their outstanding phenotype characterized by increased toxin production and higher transmissibility compared to the conventional M1 strain [13–16, 25]. In England, the prevalence of M1UK lineage increased significantly from 8.3% (3 out of 36 cases) to 84.3% (252 out of 299 cases) between 2011 and 2016 [13], and 91.1–95.7% from 2020 to 2023 [26, 27]. The isolation rate of M1UK lineage strains from iGAS infections was reported to be 29.8% for 2022–2023 in Germany [14], and 10.8% for 2019–2021 in the United States [25]. In Belgium, the total number of iGAS infections in 2022 and 2023 increased by 1.3 to 2-fold compared to 2018 and 2019, with M1UK constituting 71.6% of the *emm1* strains associated with bloodstream infection [9]. In Japan, of 377 GAS strains isolated from STSS cases since January 2024, 221 (58.6%) were M1 strains, with 194 (51.5%) classified as M1UK lineage strains [16]. Given the virulence and adaptability of the M1UK strain, there is a pressing concern that the spread of the M1UK lineage strains could lead to a further increase in iGAS infections and STSS in Japan. Clinical and epidemiological information on unreported GAS infections, other than STSS, remains scarce in Japan; however, the spread of M1UK lineage strains will continue to raise concerns as observed in international countries.

The strength of our study lies in its ability to clarify the clinical burden and characteristics of unreported GAS infections in Japan. Our findings demonstrated that the incidence of iGAS infections was twofold higher than that of notified STSS cases, with comparable mortality rates between the distinct cohorts. This observation underscores the urgent necessity to implement a more comprehensive surveillance system that incorporates iGAS cases within the Japanese national infectious disease monitoring framework. Several limitations should be noted as well. First, this is a retrospective study, and thus, clinical diagnosis relies on the discretion of the attending physician. Also, the notification of cases as Category V infectious disease depends on the diagnostic facility and clinical assessment. Consequently, the number of notified STSS cases may be underestimated. Second, information regarding all *emm*-types including the M1UK lineage strain is limited to a subset of cases notified as STSS in 2024, leaving its broader involvement in other GAS infections unclear. Further in-depth national surveillance of the M1UK lineage strain is anticipated in the future. Thirdly, the case number of GAS pharyngitis is likely substantially underestimated due to the predominant diagnostic reliance on rapid antigen detection testing in Japanese clinical settings. Fourthly, in accordance with the United States CDC criteria for confirmed iGAS cases, pneumonia was not included in the iGAS infection in this study. Fifth, we could not differentiate between community-acquired and healthcare-associated onset of the disease. Finally, the prognosis of the two patients transferred to other hospitals remains unknown, potentially resulting in an underestimation of the outcomes.

In conclusion, we highlighted that unreported iGAS cases involve patients at a twofold higher frequency compared to the notified STSS cases, despite comparable clinical outcomes. The clinical burden of unreported GAS infections warrants attention due to their high rates of surgical interventions, ICU admissions, and in-hospital mortality. Our investigation elucidated the clinical characteristics and outcomes of current GAS infections during a period of rapidly escalating incidence and disease severity. These findings have potentially significant implications for strengthening the national surveillance system for streptococcal infections in Japan.

## Data Availability

No datasets were generated or analysed during the current study.
